# Investigating the association between health vulnerabilities and police enforcement during the Covid-19 pandemic: A novel study using linked administrative data in Scotland

**DOI:** 10.1177/26338076241304446

**Published:** 2025-01-22

**Authors:** Victoria Gorton, Susan McVie, Ben Matthews, Kath Murray

**Affiliations:** 3124 School of Law, University of Edinburgh, Edinburgh, United Kingdom; 7622 Sociology, Social Policy and Criminology, University of Stirling, Stirling, United Kingdom; 3124 School of Law, University of Edinburgh, Edinburgh, United Kingdom

**Keywords:** Covid-19, health vulnerabilities, police enforcement, fixed penalty notice, justice inequality, Scotland

## Abstract

Public health regulations introduced in response to the Covid-19 pandemic placed unprecedented restrictions on the U.K. public. To maximise compliance with the regulations, new policing powers were introduced enabling officers to issue Fixed Penalty Notices (FPNs) to those believed to have breached them. In Scotland, where over 20,000 Covid-FPNs were issued for regulatory breaches, police officers reported particular challenges dealing with non-compliance amongst people with health vulnerabilities involving mental illness and substance use. Health studies suggest that people with such conditions were most severely impacted by the pandemic in a whole range of ways; however, there are no existing studies on whether this includes police use of enforcement. Our study addresses this gap using linked administrative data from police and health organisations in Scotland. Using a case-control design, we found that people who had accessed health services for psychiatric conditions or substance use were more likely to have received a Covid-FPN, especially during the first lockdown. The strength of this association was greatest amongst people with multiple health conditions and those accessing health services both before and during the pandemic. The findings suggest the new policing powers impacted disproportionately on people suffering from mental illness and/or addictions and point to a previously unidentified justice inequality. This novel administrative data linkage study highlights the importance of taking health vulnerabilities into greater consideration when planning for future pandemic preparedness.

## Introduction

On 23 March 2020, the United Kingdom (U.K.) Prime Minister announced a nationwide lockdown in response to the unfolding Covid-19 pandemic. Within days, the four devolved U.K. nations had introduced public health regulations aimed at limiting the spread of the virus. These placed unprecedented restrictions on people's everyday lives, including instructions to stay at home, avoid unnecessary travel, and socially distance from other people. Politicians and public health advisors urged people to comply with the regulations voluntarily; however, like many other countries across the world (see Maskály et al., 2021), U.K. police forces were given temporary new powers of enforcement which allowed officers to issue Fixed Penalty Notices (FPNs), and in extreme cases arrest, those who failed to comply (see Murray et al,, 2024). To limit the potential for damaging police-public relations, guidance was issued to all U.K. police forces recommending a proportionate approach to the use of enforcement. Nevertheless, over the following 2 years, more than 140,000 Covid-FPNs were issued in Scotland, England, and Wales—some as high as £10,000—for behaviours that, under normal circumstances, would have been perfectly law abiding (see Gorton et al., 2022; McVie et al., 2023).

In Scotland, interviews with frontline police officers highlighted that people with certain types of health vulnerabilities, including mental illness, drug addiction, and alcohol misuse, had particular difficulty coping with and abiding by the restrictions (HMICS, 2020, 2021). This aligns with global health studies that have revealed the deleterious effects of both the disease and public restrictions on people affected by mental illness and substance use (see Roberts et al., 2021; Sampogna et al., 2022; Serafini et al., 2020). However, to date, no studies have examined whether people with health vulnerabilities were disproportionately impacted by policing powers introduced to reinforce public health regulations. Many types of inequality have already been identified in the wake of the pandemic, including in education, the labour market, living standards, health, and the economy (see Blundell et al., 2022); however, potential justice inequalities have been largely neglected.

Our paper addresses this knowledge gap using a novel study that links individual-level administrative data on health vulnerabilities (i.e., registered contact with health services relating to psychiatric conditions, drug use, and/or alcohol misuse) with data on police enforcement (i.e., Covid-FPNs issued in response to regulatory breaches) during the pandemic in Scotland. The overarching aim of the study was to determine whether people with health vulnerabilities were at increased relative risk of receiving a Covid-FPN. Our study uses data from Scotland because scrutiny around the use of the new policing powers led to significant data sharing between Police Scotland and the study authors as part of the Policing the Pandemic in Scotland study.^1^ Various barriers have previously prevented the sharing of police administrative data for linkage-based research in Scotland (McVie, 2016); however, close working relationships established during the pandemic helped to overcome these challenges and facilitate the creation of the first bespoke police dataset for linkage to population health data. Our achievement in securing this data linkage is highly significant as, to our knowledge, equivalent data are not linkable in other parts of the U.K. and no similar study has been attempted in any other jurisdiction. We were inspired to persevere in the face of many difficulties and delays by our good friend and collaborator, Professor Anna Stewart, who understood better than most the many challenges of securing linkable justice data for research.

Before discussing the study design and findings in detail, we set out relevant literature on the Scottish policing response and the impact of the pandemic on people with health vulnerabilities.

## Literature review

### Scotland's policing response to Covid-19 in the United Kingdom context

The principle of policing by consent is at the heart of democratic models of policing and is strongly endorsed by all U.K. police forces. In line with this principle, the College of Policing (CoP) and the National Police Chiefs’ Council (NPCC) jointly issued guidance in respect of the policing response to Covid-19 (known as Operation Talla). The guidance advised officers to adopt a 4Es approach (emphasising that they engage, explain, and encourage, before resorting to enforcement) and “make sensible decisions, employ their judgement” and proceed to enforcement only “if necessary and proportionate” (CoP/NPCC, 2020, Section 3). According to an inspection report on Operation Talla in England and Wales, “all forces followed the 4Es approach in their policing of the Coronavirus regulations” and enforcement was used only as “a last resort” (HMICFRS, 2020, p. 5). This statement was, however, based on “self-assessment” as police forces in England and Wales did not record non-enforcement interactions with the public during the pandemic.

In Scotland, where policing matters are devolved to the Scottish Government, a single Police Service of Scotland (hereafter “Police Scotland”) operates independently from the other U.K. forces and is held to account by the Scottish Police Authority (SPA). In April 2020, the SPA established an Independent Advisory Group (IAG)^2^ to provide scrutiny around Operation Talla, and an evidence-led strategy was developed to examine whether policing practice was in line with human rights principles and Police Scotland's core values. Informing the work of the IAG was a bespoke reporting system created by Police Scotland, known as the Coronavirus Intervention (CVI) system, on which officers were encouraged to record the nature and extent of all relevant encounters. Between March 2020 and May 2021, the CVI system recorded over 20,000 Covid-FPNs in Scotland, which represented far greater use of police fixed penalties than normal (see McVie, 2022). However, because the CVI system collected data on all types of Covid-related interactions, it revealed that only 11.6% of all encounters involved the issue of a Covid-FPN, while a further 0.7% involved an arrest (Gorton et al., 2022). In other words, the CVI system verified that the 4Es approach helped minimise police use of enforcement in Scotland.

**Figure 1.**
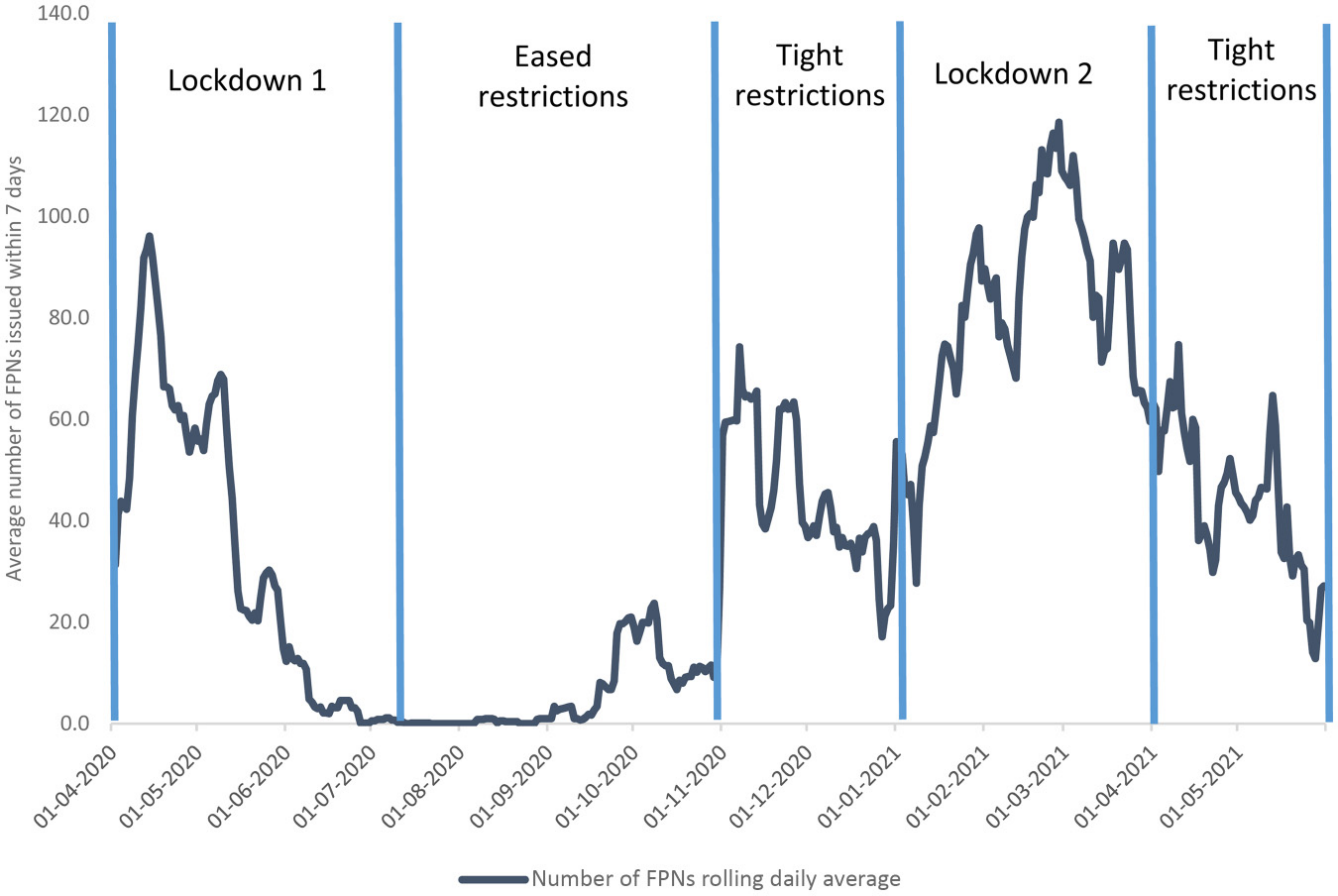
Number of Covid-FPNs issued in Scotland (April 2020–May 2021).

Figure 1 illustrates the temporal patterning of police enforcement using data from the CVI system. It shows wide temporal variation in the number of Covid-FPNs issued in Scotland, largely according to the level of restrictions in place at the time.^3^ Fining peaked sharply during the early weeks of the first lockdown, which lasted from 23 March to 10 July 2020. During this time, all non-essential shopping and travel was banned, and everyone except key workers was required to stay at home. The restrictions were eased during the summer of 2020, although some rules remained in place relating to travel and social gatherings; however, very few Covid-FPNs were issued during this period. Infection rates started to increase in autumn 2020, as students returned to schools, colleges, and universities. In response, the Scottish Government tightened restrictions in relation to social gatherings, and the number of Covid-FPNs increased. Enforcement rose sharply after the second lockdown was imposed on 5 January 2021 (due to high infection rates) and remained high until 1 April 2021, during which stay at home measures were re-introduced and travel was allowed for essential purposes only. From 2 April to the end of May 2021, travel and social gatherings continued to be subject to tight controls, although the regulations allowed greater movement within local authority boundaries. The number of Covid-FPNs issued during this final phase tailed off over time.

It is clear from Figure 1 that the likelihood of being issued with a Covid-FPN in Scotland was greatest during periods of tight restriction, especially during the lockdowns. In interviews conducted just after the first lockdown, frontline officers commented that most people were compliant, but individuals with health vulnerabilities had particular difficulty coping with the restrictions, especially those with pre-existing conditions. Officers described “instances of people with mental health issues who were finding it difficult to cope with lockdown, as they were not seeing friends or family and not having the same access to support mechanisms” (HMICS, 2020, p. 5). In addition, substance users were described as vulnerable, having complex needs and lacking adequate support from partner agencies. One community officer talked about “engaging on a daily basis with around 50 individuals with complex needs, including substance misuse and addictions, which did not change due to the pandemic” (HMICS, 2020, p. 7).

In follow-up interviews conducted after the second lockdown, officers described a distinct change in operational policing challenges, compared to the first lockdown. Overall compliance had “waned over time”—especially amongst younger people—and there was “a greater incidence of individuals interpreting the rules to suit their own ends” (HMICS, 2021, p. 3). Nevertheless, officers continued to note that people suffering from mental illness found it more difficult to comply and felt socially isolated without access to the usual support networks. There was also an increase in calls for service classified as cause for concern, “many of which were associated with mental ill health” (HMICS, 2021, p. 5). These insights from the police interviews in Scotland align with international research evidence on the impact of the pandemic—and the restrictions imposed as a result of it—on people suffering from mental illness and substance use.

### The impact of the pandemic on health vulnerabilities

The negative impact of Covid-19 on mental health has been well documented globally. In an early literature review, Serafini et al. (2020) concluded that the spread of Covid-19 had caused loneliness, fear, anxiety, frustration, and boredom which reduced subjective wellbeing and resulted in an increase in psychiatric conditions within the general population. Moreover, the rise in psychological distress caused by Covid-19 was linked to increases in problematic substance use, including both alcohol and drugs (see Chacon et al., 2021; DiClemente et al., 2021; Roberts et al., 2021; Schecke et al., 2022). Importantly, research suggests that while the pandemic increased the prevalence of these issues across the general population, it had a particularly detrimental effect on those with pre-existing conditions relating to mental illness (Sampogna et al., 2022) and addictions (Fabelo-Roche et al., 2021), including poly drug users (Avena et al., 2021), and those with multimorbidity involving both psychiatric and substance use disorders (Marel et al., 2021).

There is no evidence that mental ill health and/or substance use were the universal drivers of regulatory non-compliance—many other factors have been identified (see Nivette et al., 2021; Tomlinson et al., 2022; Wright et al., 2021). However, a few studies indicate that such conditions played a contributing role. Wright et al. (2022) reported that individuals whose mental health deteriorated during the pandemic were less likely to report being compliant than those whose mental health stayed the same, while Maekelae et al. (2020) suggested that heightened mental stress reduced the effectiveness of governmental messaging around restrictions and, thus, increased non-compliance. An international comparative study also found that substance use had become a maladaptive coping strategy during the pandemic which was associated with increased non-compliance (Kleitman et al., 2022). Similarly, Acuff et al. (2021) noted that Covid restrictions increased substance-related non-compliance due to fewer constraints on using substances and reduced access to substance-free alternatives. Feelings of loneliness and social isolation and lack of access to outdoor space also contributed to symptoms of stress, anger, and confusion amongst vulnerable groups (Burton et al., 2023).

Within the U.K., many types of inequality are known to have been exacerbated by Covid-19 (see Blundell et al., 2022); however, potential justice inequalities have largely been overlooked. Despite many studies on the severe impact on people with health vulnerabilities, no one has yet examined whether this resulted in an increased propensity to come into conflict with law enforcement. This is important because many countries used monetary penalties to bolster regulatory compliance during the pandemic (Maskály et al., 2021), yet such sanctions are known to have a disproportionate and unequal impact on the population (see Murray et al., 2024).

## A novel data linkage study

In order to address this gap in knowledge, our study examines the association between police enforcement and health vulnerabilities during the Covid-19 pandemic using linked administrative data. It focuses on Scotland because the work of the IAG provided a window of opportunity for the study authors to negotiate access to a dataset containing information on all Covid-FPNs issued by Police Scotland. Historically, there have been many barriers to the sharing of justice data for research in Scotland (McVie, 2016), which align closely with those discussed by Stewart et al. (2015) in the Australian context. However, close working relationships established during the pandemic helped to overcome these challenges and facilitate the creation of a bespoke police dataset that could be used for linking to Scotland's rich health data. It is currently impossible to conduct an equivalent study for other parts of the U.K. as the necessary data cannot be linked.

### Study aim and research questions

The overarching aim of the study was to determine whether there was a significant association between health vulnerabilities (including mental illness, drug use, and alcohol misuse) and police use of enforcement in Scotland during the pandemic. To achieve this aim, our study was guided by three research questions:

1. How did the profile of health vulnerabilities amongst Covid-FPN recipients compare to a matched group from the general population?
2. To what extent did the comorbidity of health vulnerabilities increase the likelihood of receiving a Covid-FPN?
3. To what extent did the timing of health service contact increase the likelihood of receiving a Covid-FPN?

Given that police officers had noted distinct differences in the profile of those that presented challenges during the first and second lockdown, we also explored differences in the results for all three questions between these two periods.

### Study design and ethics

The study involved linking individual-level administrative data from Police Scotland and Public Health Scotland (PHS), as well as other geographical and property-related data sources. The dataset provided by Police Scotland contained details of all Covid-FPNs issued in Scotland, while PHS provided information on health service contact relating to psychiatric conditions, drug-related problems, and alcohol-related conditions. The research was undertaken within Scotland's National Safe Haven (NSH), one of several United Kingdom-based trusted secure environments which enable pseudonymised data to be linked under strict legal and ethical conditions.^4^ This aspect of the research was supported by the Scottish Centre for Administrative Data Research (SCADR).^5^

Due to the sensitive nature of the datasets, extensive consideration was given to ethics, permissions, and approvals prior to data linkage. An ethics application and a Data Privacy and Impact Assessment (DPIA) were approved by the University of Edinburgh. Formal permission for use of the Covid-FPN data was obtained from Police Scotland, and approval for use of the health data was obtained from Scotland's Health and Social Care Public Benefit and Privacy Panel (HSC-PBPP). Police Scotland and PHS also carried out DPIAs to ensure that the data sharing was legal and proportionate. Data Processing Agreements were established to ensure legal gateways for data sharing between all parties involved in the data linkage process (see below); and the privacy notices for Police Scotland and PHS were checked to ensure transparency around sharing of data for research purposes. The research team undertook accredited Safe Researcher training and signed User Agreements governing ethical and secure use of the linked datasets.

A “case-control” design was adopted for the study. Case-control studies are common in epidemiology as a way of helping to determine if exposure to a specific condition is associated with a particular outcome, especially when that outcome is rare (Lewallen & Courtright, 1998). Such studies are less costly and time-consuming than prospective longitudinal population studies, and they require far less data (Breslow, 2005). The focus of our analysis was to determine whether exposure to particular health vulnerabilities was associated with receiving a Covid-FPN; therefore, our study involved identifying “cases” (people living in Scotland who received a Covid-FPN) and matching them to a group of “controls” (people living in Scotland with a similar profile to the cases but who did not receive a Covid-FPN). For each case, three controls were matched based on demographic characteristics known to be significantly associated with enforcement (based on the work of Gorton et al., 2022).

### Data sources

Police Scotland provided management data for all Covid-FPNs issued under Scotland's public health regulations between 27 March 2020 and 31 May 2021. This included date and time of FPN issue; police division of issue; and basic demographic information (age, sex and, in some cases, ethnicity) for the person to whom it was issued. Individual identifiers allowed multiple Covid-FPN recipients to be identified; however, for the purposes of this paper, each case represents one individual who received at least one fine (using only the first fine for those who received more than one).

PHS provided data from five health systems: hospital inpatient and day cases (SMR01); psychiatric inpatient and data cases (SMR04); emergency department attendance (A&E data); Scottish Drugs Misuse Database (SDMD); and Scottish Ambulance Service (SAS). Records covering the period from 1 March 2019 to 31 May 2021 were used, allowing us to examine health service contact in the year prior to the pandemic as well as contact during the pandemic. These data sources provided the key independent variables for analysis, as well as demographic information about the matched controls.

Additional information about household size was obtained from the CURL (CHI-UPRN Residential Linkage) dataset (see Clark & Dibben, 2020). Information on property type was acquired from the AddressBase dataset, accessed via the Ordinance Survey, which was linked using Unique Property Reference Numbers (UPRN) codes as recorded by CURL. Explanation of these variables is provided below.

### Data linkage process

A dataset containing Personal Identifiable Information (PII) (including name, date of birth and address) for all people in receipt of a Covid-FPN (*N* = 20,410) was shared by Police Scotland with the National Records of Scotland (NRS). NRS acted as a trusted third party between Police Scotland and PHS to minimise risk of individual identification. Using probabilistic matching, NRS linked as many FPN recipients as possible (*n* = 16,370) to the Community Health Index (CHI) number (the unique identifier held by PHS for those who have had contact with health services). NRS created a new Unique Reference Number (URN) which was shared with both Police Scotland (attached to the PII) and PHS (attached to the CHI).^6^ Police Scotland then shared the FPN payload data with PHS including the new URN, which was used by PHS to link to the relevant health datasets via the associated CHI.

Matching for the case-control design was undertaken by the Electronic Data Research and Innovation Service (eDRIS) team at PHS. Three randomly matched controls per FPN recipient (*n* = 45,808) were drawn from the CHI population based on personal characteristics (age and sex) and residential profile (Scottish Index of Multiple deprivation decile and Local Authority). This process used exact matching, with the exception of age where there was a 1 year tolerance between cases and controls. Once the matching was complete, all datasets were pseudonymised (i.e., PII and CHI numbers were removed) by the eDRIS team before being provided to the researchers via Scotland's NSH.

For reasons of space, details of the matched profile of the cases and controls are not presented here.^7^ Analysis showed that the two groups were matched very closely; however, compared to the general population, both groups substantially over-represented younger people, males, and those living in the top quintile of deprivation based on the SIMD. The matched cases and controls also over-represented people living in local authorities in the west of Scotland. These differences to the population reflect known disproportionality in police fining rates (see Gorton et al., 2022).

### Variables used in analysis

#### Outcome variable

Our outcome of interest was the binary measure indicating whether each individual had received a Covid-FPN (case) or not (control).^8^

#### Independent variables

Three types of health vulnerability measures were derived from records of health service contact relating to any psychiatric, drug-related, or alcohol-related condition (based on International Classification of Diseases, Tenth Revision (ICD-10) codes) drawn from all five health datasets (SMR01; SMR04; A&E; SDMD; and SAS) during the study period. First, binary measures indicating whether people had accessed services for each type of health vulnerability (scored 1) or not (scored 0) were created. Second, an ordinal measure represented the number of types of health vulnerability for which each person had accessed services (i.e., a measure of multimorbidity). Third, a nominal measure was created based on the dates of health service contact, allowing us to assess whether likelihood of police enforcement varied according to duration and timing of health vulnerabilities.

#### Control variables

Two control variables were included in the analysis based on police observations about the potential exacerbating effect on health vulnerabilities of loneliness/social isolation and lack of access to greenspace (HMICS, 2020, 2021). A binary variable was constructed from the CURL dataset identifying whether the individual was living in a single person household, thus providing a proxy for social isolation. Another binary variable was constructed from the AddressBase dataset identifying people who were in a self-contained flat (including maisonettes/apartments) which provided a proxy for lack of access to a garden or private greenspace.^9^

### Statistical methods

Research question 1 was addressed using simple descriptive analysis (comparing the matched groups), while conditional logistic regression analysis was used for research questions 2 and 3. This type of modelling is a standard method for case-control analysis as it accounts for the matched nature of the dataset (Kuo et al., 2018). While standard advice in social sciences is to interpret logistic regression results using marginal effects (Breen et al., 2018), there is no straightforward way to do this using conditional logistic regression. Instead, as is typical for a case-control design (Breslow, 2005), we report the odds ratios, *p*-values and confidence intervals from each model. Stata 16 was used for all analysis.

## Results

### Descriptive analysis

Table 1 addresses research question 1 by summarising the prevalence, comorbidity, and timing of health service contact for the matched groups. It confirms that Covid-FPN recipients were more likely than their matched controls to have accessed health services for a psychiatric, drug-related, and/or alcohol-related condition between March 2019 and May 2021. Almost one in five (18.6%) Covid-FPN recipients had accessed relevant health services compared with less than one in 20 (3.9%) of the control group, reflecting a disparity of almost five times. Table 1 also shows that, compared to the controls, Covid-FPN recipients were more likely to have had health service contact for all three types of health vulnerability, but especially drug and alcohol-related conditions.

**Table 1.** Comparing the prevalence, comorbidity, and timing of health service contact^a^ for Covid-FPN recipients and matched controls, Scotland.

	Cases (Covid-FPN recipients)	Controls (matched individuals with no Covid-FPN)	Disparity rate (cases/controls)
Health service contact for any of the three conditions	18.6%	3.9%	4.8
Health service contact for:			
Drugs conditions	12.4%	1.8%	6.9
Alcohol conditions	11.4%	1.7%	6.7
Mental health conditions	10.4%	2.4%	4.3
Health service contact for:			
No conditions	81.4%	96.1%	0.8
One condition	7.7%	2.5%	3.1
Two conditions	6.1%	1.0%	6.1
Three conditions	4.7%	0.5%	9.4
Timing of contact (amongst those with contact only):			
Contact only before pandemic	22.4%	38.9%	0.6
Contact only during pandemic	27.1%	32.8%	0.8
Contact both before and during	50.5%	28.3%	1.8
Number of individuals	16,370	45,808	

a Health service contact measured between 1 March 2019 and 31 May 2021.

Looking at the number of types of conditions for which people had sought support from services, the cases were also more likely than the controls to have experienced comorbid health vulnerabilities. In fact, Covid-FPN recipients were six times more likely to have accessed services for two types of health condition, and nine times more likely to have sought support for all three conditions, than the control group. The final section of Table 1 shows the timing of health service contact. Covid-FPN recipients were almost twice as likely as the equivalent controls to have accessed services both before and during the pandemic, while those in the control group were more likely than the cases to have had contact only before or during the pandemic.

**Figure 2.**
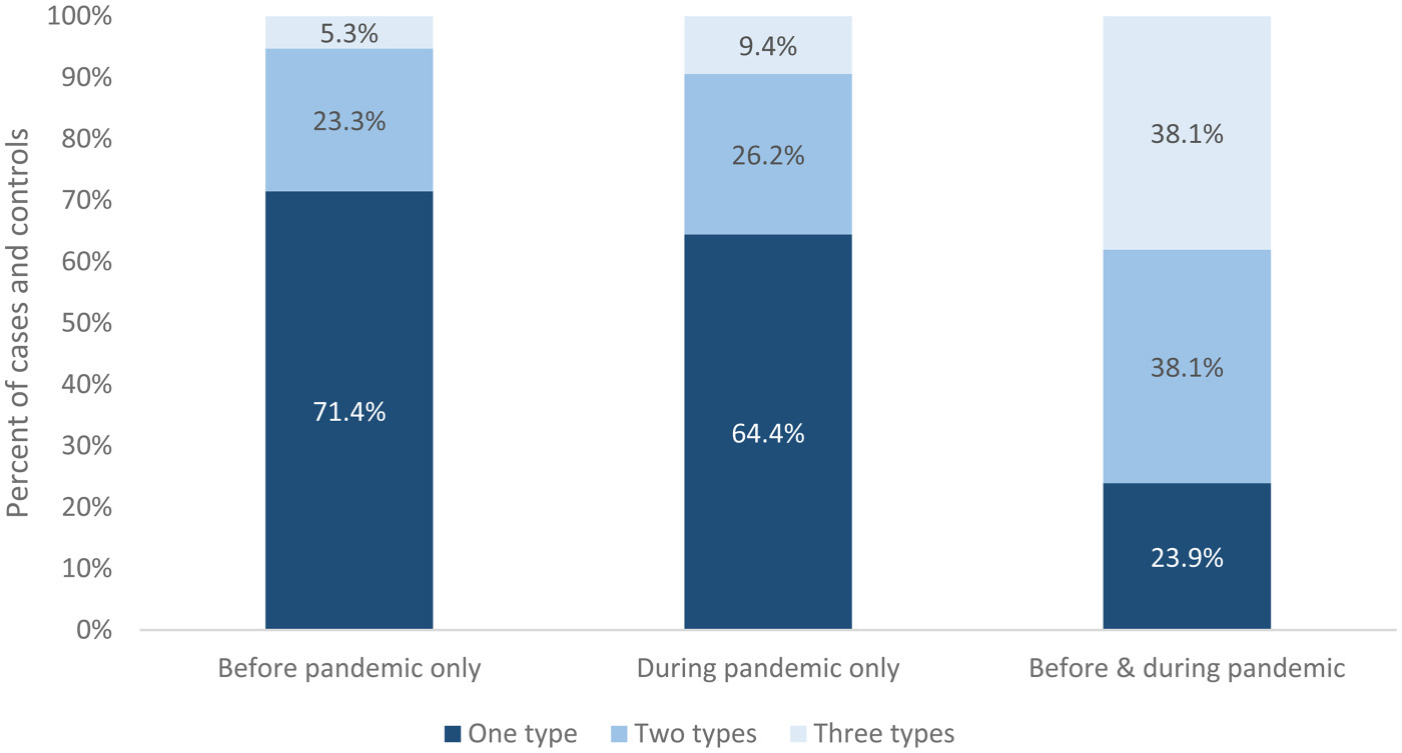
Percentage of people with different types of health condition by timing of health service contact in Scotland.

*Note*. Before pandemic only = 1 March 2019 to 26 March 2020; during pandemic only = 27 March 2020 to 31 May 2021; before and during pandemic = 1 March 2019 to 31 May 2021.

Unsurprisingly, there was a close relationship between the number of health conditions experienced and the timing of service contact, as illustrated in Figure 2. Amongst those who accessed services only before or only during the pandemic, the majority (71.4% and 64.4%, respectively) were known to have sought support for only one type of condition. However, amongst those who accessed services both before and during the pandemic, only 23.9% sought support for one type of condition, whereas equal proportions (38.1%) had sought support for two or three types of health vulnerability. Those who were long-standing service users were, therefore, more likely to have multimorbid conditions.

The results from Table 1 and Figure 2 provide strong justification for examining both the profile and timing of known health vulnerabilities for those who were subject to police enforcement during the pandemic. Nevertheless, it is worth noting that the majority of people in both groups were not known to health services for any of the three health conditions. As noted earlier, therefore, patterns of police enforcement under the Covid-19 regulations are likely to have been associated with a wider range of factors.

For reasons of space, the descriptive information for the two control variables is not presented here.^10^ However, relatively little difference was found between the cases and controls in terms of household size or property type. Covid-FPN recipients were slightly (albeit significantly) more likely than the matched controls to have been living in a single person household during the pandemic, so involvement in illegal social gatherings due to loneliness or isolation may have increased the likelihood of some people coming into contact with the police. In addition, Covid-FPN recipients were slightly (but significantly) more likely than the matched controls to be living in a self-contained flat or apartment during the pandemic, so leaving home to access outdoor space during periods of restriction may have been a factor for some in attracting police attention. It is impossible to be certain of the reason for police contact based on these data as this information was not provided; however, the differences between groups provides some justification for including these two variables in the analysis to reduce potential confounding.

To examine change over time in these associations, Table 2 compares the prevalence, comorbidity, and timing of health service contact for individuals who received Covid-FPNs during the first or second lockdown period and their matched controls. Cases were more likely than controls to have accessed health services for one or more types of vulnerability during both lockdown periods; however, the degree of disparity between the matched groups from the first lockdown was much larger than that from the second. Almost 40.0% of those who received a Covid-FPN during the first lockdown had sought health service support during the study period (eight times greater than their matched controls); however, this reduced to 15.0% for those fined during the second lockdown (four times greater than the matched controls). Similarly, while cases were more likely than controls to have experienced comorbidity of health conditions during both lockdown periods, the degree of disparity between groups was far greater during the first lockdown than the second. For example, 12.0% of Covid-FPN recipients during the first lockdown accessed health services for all three types of health vulnerability (20 times greater than the matched controls), whereas the equivalent figure for the cases during the second lockdown was 3.4% (almost seven times greater than the matched controls).

**Table 2.** Comparing the prevalence, comorbidity, and timing of health service contact^a^ for Covid-FPN recipients during lockdown 1 (27 Mar–10 Jul 2020) and lockdown 2 (5 Jan–1 Apr 2021) and matched controls, Scotland.

	Lockdown 1			Lockdown 2		
	Cases (Covid-FPN recipients)	Controls (matched individuals with no Covid-FPN)	Disparity rate (cases/controls)	Cases (Covid-FPN recipients)	Controls (matched individuals with no Covid-FPN)	Disparity rate (cases/controls)
Health service contact for any of the three conditions	39.5%	4.8%	8.2	15.0%	3.8%	3.9
Health service contact for:						
No conditions	60.5%	95.2%	0.6	85.0%	96.2%	0.9
One condition	13.6%	2.8%	4.9	6.7%	2.4%	2.8
Two conditions	13.9%	1.4%	9.9	4.9%	0.9%	5.4
Three conditions	12.0%	0.6%	20.0	3.4%	0.5%	6.8
Timing of contact (amongst those with contact only):						
Contact only before pandemic	17.9%	33.7%	0.5	24.6%	39.5%	0.6
Contact only during pandemic	28.2%	45.9%	0.6	40.5%	50.4%	0.8
Contact both before and during	60.1%	34.9%	1.7	46.5%	27.0%	1.7
Number of individuals	3129	8559		7478	20,942	

a Health service contact was measured between March 2019 and May 2021.

Looking at the timing of health service contact, for those who had accessed relevant services at least once during the whole study period, there was little change in the disparity rate between cases and controls from lockdown 1 to lockdown 2. For example, during both lockdowns, the cases were 1.7 times more likely than the controls to have contacted services both before and during the pandemic. There was some change in the timing of health service contact within groups over the two lockdowns (i.e., the percentage of cases and controls who had service contact both before and during the pandemic was higher in lockdown 1 than lockdown 2). However, the disparity rates suggest that the temporal nature of service contact changed in a similar way for both cases and controls.

Overall, the prevalence of health service contact was very similar for the control groups during the first and second lockdown periods; however, there was a large decline in contact amongst the cases. This suggests a distinct shift over time in the overall profile of those who were fined, reflecting a much lower concentration of people with health vulnerabilities. It is highly likely that this reflects a broadening of police interactions with a wider spectrum of groups within the population (as suggested by Gorton et al., 2022 and HMICS, 2021) due to the much larger number of people fined during the second lockdown (7,500 compared to just over 3,000 in the first). Nevertheless, it is clear that people with health vulnerabilities continued to be disproportionately represented amongst those fined under the Covid-19 regulations as the pandemic progressed.

### Regression analysis

Conditional logistic regression analysis was used to examine the strength of association between health vulnerabilities and police enforcement during the pandemic while adjusting for the two control variables. Table 3 addresses research question 2 by showing results for three models, using number of types of health vulnerability as a measure of comorbidity. Model 1 looks at results across the whole pandemic, while models 2 and 3 only include individuals fined during the first and second lockdowns (respectively) and their matched controls. The odds ratios for all three models are highly significant, suggesting that even with a case-control design, and taking account of other potential confounding factors, being known to health services during the study period for one or more of these types of health vulnerability was positively associated with being in the Covid-FPN group. Moreover, the more types of health condition recorded in administrative records, the larger the odds of being in the Covid-FPN group.

**Table 3.** Conditional logistic regression based on number of types of health vulnerability (dependent variable = case-control membership across three time periods).^a^

	Model 1			Model 2			Model 3		
	27 Mar 2020–31 May 2021			27 Mar–10 Jul 2020			5 Jan–1 Apr 2021		
	Odds ratio	95% CI	*p*	Odds ratio	95% CI	*p*	Odds ratio	95% CI	*p*
Number of types of condition recorded:									
One	3.68	3.37–4.01	<.001	7.43	6.14–9.00	<.001	3.12	2.74–3.56	<.001
Two	7.16	6.35–8.08	<.001	14.75	11.60–18.77	<.001	5.86	4.86–7.06	<.001
Three (reference = none)	11.97	10.18–14.09	<.001	32.27	22.99–45.31	<.001	7.77	6.08–9.93	<.001
One person household (reference = other household size)	1.25	1.19–1.32	<.001	1.51	1.33–1.70	<.001	1.23	1.14–1.33	<.001
Self-contained flat (reference = other property type)	1.21	1.15–1.28	<.001	1.46	1.28–1.67	<.001	1.22	1.13–1.33	<.001
	*n* = 61,079 Pseudo *R*^2 ^= .0753			*n* = 11,413 Pseudo *R*^2 ^= .2414			*n* = 27,923 Pseudo *R*^2 ^= .051		

a Time periods are based on when Covid-FPNs were issued. Health service contact was measured between March 2019 and May 2021.

Model 1 shows that, over the course of the study period, individuals with multimorbidity had 12 times greater odds than someone with no known health conditions of being in the Covid-FPN group rather than the matched controls. However, this figure was much larger in model 2, representing the first lockdown, where the equivalent odds were 32. It is notable that the odds ratios for those with one or two types of health condition in model 2 were double those in model 1, while the odds ratios for those with all three types of health condition were almost three times higher in model 2. Model 3, which represents the second lockdown, estimated smaller odds ratios for the number of health conditions than model 1; nevertheless, the odds were still highly significant and could be considered moderate to large in terms of effect size (see Chen et al., 2010).

To address research question 3, Table 4 presents three conditional logistic regression models using timing of health service contact as the independent variable of interest. Like Table 3, the odds ratios for all three models are highly significant which confirms that any health service contact (no matter when it took place) was associated with being in the Covid-FPN group. The most interesting finding from models 4 to 6 is that, compared to those with no health service contact, individuals who were in contact with health services both before and during the pandemic were by far the most likely to be in the Covid-FPN group rather than the matched controls. Across the pandemic as a whole, the relevant odds ratio was almost 10 (model 4); however, this more than doubled to 22.5 when looking just at the first lockdown period (model 5). People who accessed health services during the pandemic but not in the year before it had slightly higher odds of being in the Covid-FPN group than those with health service contact in the year prior to the pandemic but not during it (this was true across all three time periods). These findings suggest that people with pre-existing health vulnerabilities who needed ongoing access to services during the pandemic had the highest relative likelihood of being fined by the police; however, there was also an elevated risk amongst those who may have developed health conditions relating to mental illness or addictions during the course of the pandemic, as well as those with known prior issues.

**Table 4.** Conditional logistic regression based on timing of health service contact (dependent variable = case-control membership across three time periods).^a^

	Model 4			Model 5			Model 6		
	27 Mar 2020–31 May 2021			27 Mar–10 Jul 2020			5 Jan–1 Apr 2021		
	Odds ratio	95% CI	*p*	Odds ratio	95% CI	*p*	Odds ratio	95% CI	*p*
Timing of contact									
Before pandemic only	3.30	2.95–3.69	<.001	7.19	5.60–9.22	<.001	2.88	2.43–3.42	<.001
During pandemic only	4.56	4.07–5.10	<.001	8.15	6.40–10.36	<.001	3.78	3.19–4.48	<.001
Before and during pandemic (reference = no contact)	9.81	8.77–10.97	<.001	22.48	18.01–28.07	<.001	7.21	6.07–8.57	<.001
One person household (reference = other household size)	1.25	1.18–1.31	<.001	1.51	1.33–1.70	<.001	1.22	1.13–1.32	<.001
Self-contained flat (reference = other property type)	1.21	1.15–1.28	<.001	1.46	1.28–1.67	<.001	1.22	1.13–1.33	<.001
	*n* = 61,079 Pseudo *R*^2 ^= .0754			*n* = 11,413 Pseudo *R*^2 ^= .241			*n* = 27,923 Pseudo *R*^2 ^= .051		

a Time periods are based on when Covid-FPNs were issued. Health service contact was measured between March 2019 and May 2021.

Interestingly, the control variables for household size and property type both had small, but significantly positive, odds ratios for all six models. This adds some credibility to the suggestion that, even taking account of people's health vulnerabilities, social isolation or loneliness (as a consequence of living alone) and lack of access to private outdoor space (as a result of living in a self-contained housing unit such as a flat) may have increased some people's risk of being fined under the Covid-19 regulations amongst this matched group of individuals, especially during the first lockdown. However, it would require much better data to explore the effect of these types of situation more robustly.

## Discussion

In the absence of an immediate pharmaceutical solution to contain the spread of, and reduce the harm caused by, Covid-19, it is unsurprising that many governments relied on the police to enforce restrictions on movement, travel and association (see Ivkovic et al., 2024). In a study of 27 countries across the world, including China, Australia, the United States of America, and parts of Europe, South America, and Africa, Maskály et al. (2021) found that 80% of policing organisations had introduced new powers of enforcement under Covid-19 administrative laws, including a combination of formal warnings, fines, and arrests. In the U.K., the decision to use FPNs as the primary sanction for regulatory breaches has never been fully explained or justified; however, it is recognised as a highly unequitable penalty that impacts disproportionately on those from more disadvantaged backgrounds (see Murray et al., 2024). Prior research shows that certain groups in society were more likely than others to receive Covid-FPNs compared to their population share across the U.K. nations, including younger people (up to age 25); males; those from minority ethnic groups; and individuals living the most deprived communities (Gorton et al., 2022; McVie et al., 2023). However, this is the first study to show that people with underlying health vulnerabilities were also disproportionately likely to receive fines under the U.K's Covid-19 regulations.

The paper fills an important gap in knowledge about the association between health vulnerabilities and police use of enforcement during the pandemic. It also exposes the exacerbation of a pre-existing problem. Long before Covid-19 hit the U.K., dealing with people in crisis as a result of mental distress or other health vulnerabilities had become a significant source of demand for policing services and “an exemplar of everyday work” for police officers (Crawford, 2024, p. 437). The known scale and severity of this demand is typically underestimated in policing data (Langton et al., 2021); however, it is argued that the capacity of police forces to deal with incidents involving some form of welfare concern has become severely stretched through a combination of austerity, leading to cuts in services and personnel within both policing and health, and service reform driven by a decentralisation agenda which weakened local governance and partnership working (Solar & Smith, 2021). It is not surprising, therefore, that the immediate impact of the Covid-19 restrictions, which led to a sharp reduction in health care services, 24 hr care and day care services (Byrne et al., 2021), was most acutely felt by police officers.

Many other studies have highlighted the detrimental impact of Covid-19 and its associated restrictions on those suffering from health vulnerabilities (e.g., Roberts et al., 2021; Sampogna et al., 2022; Serafini et al., 2020). However, our novel data linkage study is the first to show that people with a known history of psychiatric, drug-related, or alcohol-related conditions were also at elevated risk of being sanctioned by the police at a time when access to health and other support services was severely diminished. Of significant note is that the likelihood of receiving a Covid-FPN was substantially increased for people with multimorbid conditions and those with pre-existing health vulnerabilities which continued during the pandemic. These findings suggest that, despite efforts by police officers in Scotland to use the 4Es approach and minimise the use of enforcement (HMICS, 2020, 2021), people with mental health conditions and/or substance use issues were considerably more likely to meet this threshold than other similar individuals. The stark reduction in the odds of vulnerable individuals being fined between the first and second lockdown periods may be explained, at least in part, by the opening up of health services as the pandemic progressed, and the introduction of local mental health protocols and a “Mental Health Pathway” to access advice and support from specialist practitioners in Scotland (HMICS, 2021). Nevertheless, the over-representation of individuals with health vulnerabilities amongst those who were sanctioned was evident during both lockdown periods, which highlights the consequences—for both the public and the police—of adopting an enforcement-based model of policing in the context of a public health emergency.

In addition to the many other types of inequality experienced during the pandemic (see Blundell et al., 2022), we argue that our findings highlight a potential justice inequality. That the rapid introduction of tight restrictions would likely impact disproportionately on some of the most vulnerable groups in society should have been anticipated by law-makers. Likewise, the implications of empowering police officers to use enforcement in situations where potentially vulnerable people were felt to be in breach of the law should have been properly considered. However, to date, we have found no evidence to show that the Scottish or U.K. governments conducted an Equality Impact Assessment in respect of the decision to use FPNs (see McVie, 2024). Indeed, there is no available evidence that significant concerns were raised by any of the U.K. nations about using a police enforcement model. Our finding that the association between health vulnerabilities and police enforcement was consistently stronger during the first lockdown, when there was particular confusion about the implementation of the new laws (Hickman et al., 2020) and many specialist services that support vulnerable individuals were withdrawn (Lenhard et al., 2023), highlights the need for equality assessments as early as possible in any future public health emergency where restrictions are planned.

It is important to note that our analysis does not test whether policing practice itself was unjust or discriminatory. The appropriateness of any policing response would need to be considered in the context of a range of other factors, including measured differences in prevalence of non-compliance within the population; variation in public reporting and police response practices; the patterning of police patrols and localities of those with health vulnerabilities; and divergence in local policing challenges during the pandemic. From an operational point of view, scrutiny undertaken by the SPA's IAG found that policing practice was in line with human rights principles and Police Scotland values (Scott, 2022). Moreover, analysis of data from Police Scotland's CVI System indicated that officers adopted the proportionate 4Es approach recommended by the U.K. College of Policing (Gorton et al., 2022). Nevertheless, this study suggests that reliance on law enforcement to augment governmental regulations may have resulted in justice inequalities for some of the most vulnerable members of society.

### Study limitations

In recent years, the U.K. has made significant strides forward in terms of data sharing and linkage for research purposes; however, there continue to be many barriers which prevent progress in this type of work (Office for Statistics Regulation, 2024). Working closely with Police Scotland during the pandemic enabled the authors of this paper to secure a unique individual-level policing dataset and bring it together with health data for the first time in Scotland. This would not have been possible without the existence of Scotland's NSH acting as a trusted research environment, which is a model of good practice for other countries. Nevertheless, as with all studies, there are limitations to what we could achieve and what conclusions can be drawn from this study.

Our Covid-19 FPN dataset was limited in content, and no information was provided about the reasons for issuing a fine or the circumstances in which this occurred. So, while we can say with reasonable confidence that there was a significant association between health vulnerabilities and police use of enforcement, we cannot say whether, or how, people's health conditions directly contributed to police officer decision making during encounters. In addition, our health vulnerability measures were based on data from formal health systems (including ambulance, emergency room, and hospital data), but many individuals who suffer from mental ill health or substance abuse many never seek support from these organisations. Moreover, access to such services was restricted during the pandemic, so we may be under-recording health vulnerabilities that occurred at that time. General practitioner data may have yielded further information, but that is not currently linkable at population level in Scotland.

In terms of coverage, we were able to study the profile of approximately 80% of those who received a Covid-FPN in Scotland. The remaining cases could not be matched by the National Records of Scotland using a CHI number. In many cases, this was because the person fined was not a Scottish resident (and had, most likely, been fined for entering Scotland while cross-border travel restrictions were in place). In other cases, the quality of the PII data was insufficient to provide a good match; we know that this is likely to have been the case for many individuals who were repeatedly fined. In theory, sensitivity analysis can be used to identify possible bias arising from this type of problem; however, this is a developing field (see Doidge & Harron, 2019) and the necessary software to address this problem is not available in Scotland's NSH. While this may limit the robustness of our study to some extent, we do not feel it has invalidated the key findings.

As with all case-control studies, the quality of the matches is critical (see Paixao et al., 2017). In our case, prior analysis of police administrative data identified a range of factors known to be associated with enforcement on which we could match; however, we were unable to account for some known confounders (such as ethnicity or prior criminal record for which insufficient data was available) and others that were suspected but unobserved (for example, household income). We are also unable to determine the impact of the 80% matching rate by NRS. These are limitations of administrative data research generally, as the extent and coverage of the data is not designed for research purposes, and something that should always be acknowledged.

Finally, our study only includes data for Scotland and it is impossible to know whether, or to what extent, the findings may be generalisable to other parts of the U.K., or other countries that adopted an enforcement-based policing model, during the Covid-19 pandemic. Nonetheless, analysis of Covid-FPN data for Scotland (Gorton et al., 2022) and England and Wales (McVie et al., 2023) produced very similar results based on the demographic profile of those who were fined. International evidence on the deleterious impact of the pandemic on mental health^11^ and substance use^12^ also provides some basis for expecting similar results in other jurisdictions. Given the widespread implementation of Covid-19 policing strategies that involved fining or another type of sanction (Maskály et al., 2021), some of which resulted in significant human rights violations (Amnesty International, 2020), it is possible that our study points to a global justice inequality. Comparage data linkage studies in other countries would be needed to establish this.

## Conclusion

This novel administrative data linkage study has helped to illuminate a significant association between chronic and long-standing health vulnerabilities and experience of police enforcement during the Covid-19 pandemic. The decision by U.K. law-makers to introduce new policing powers allowing FPNs to buttress legal restrictions has been criticised due to inherent inequity in the punishment effects of fixed financial penalties in general (Murray et al., 2024) and concern that their use during the pandemic may have been out of line with the Rule of Law (Bingham Centre, 2024). At the time of writing, the U.K. and Scottish Covid-19 inquiries are ongoing and it remains to be seen what recommendations will be made in respect of future legislative intervention. In support of such considerations, we recommend that the potential impact of any restrictions and regulations on those already experiencing, and those likely to experience, health vulnerabilities should be given serious consideration when planning for future pandemic preparedness.

## Notes

1. The Policing the Pandemic in Scotland study was funded by U.K. Research and Innovation (UKRI) under grant number ES/W001845/1. See https://www.law.ed.ac.uk/research/research-projects/policing-the-pandemic

2. The terms of reference of the IAG can be found on the Scottish Police Authority website: https://www.spa.police.uk/spa-media/o2qpcff3/draft-intro-28-4-20-1-1.pdf

3. Note that periods of lockdown and tightened restrictions varied between Scotland and parts of the United Kingdom as separate sets of public health regulations were produced by the devolved nations.

4. For further information about Scotland's NSH, see https://edinburgh-international-data-facility.ed.ac.uk/services/safe-haven-services/scottish-national-safe-haven

5. For further information about SCADR, see https://www.scadr.ac.uk/

6. The linkage rate was 79.4%. Most likely reasons for non-linkage include the individual not being a Scottish resident and poor matching due to common names and/or dates of birth or data input errors. All linked individuals were aged 16 or older.

7. Details on the profile of the matched cases and controls can be obtained by contacting the authors.

8. While some individuals were issued with more than one FPN, there were insufficient numbers to warrant the creation of an ordinal measure.

9. When people had moved address during the pandemic, the one the individual was living in at the time of receiving a fine or (for those who were not fined) the first address listed was used.

10. Descriptive information can be obtained by contacting the authors.

11. See World Health Organization: https://www.who.int/news/item/02-03-2022-covid-19-pandemic-triggers-25-increase-in-prevalence-of-anxiety-and-depression-worldwide

12. See National Institute on Drug Abuse: https://nida.nih.gov/research-topics/covid-19-substance-use
